# Enhanced Gas Classification in Electronic Nose Systems Using an SMOTE-Augmented Machine Learning Framework

**DOI:** 10.3390/s26020714

**Published:** 2026-01-21

**Authors:** Minqiang Li, Chenxi Wu, Zhiyang Wang, Zhijian Wu, Wei Huang, Junru Chen, Kaibo Yu, Ting Wen, Hongbo Yin, Zhuqing Wang

**Affiliations:** 1 School of Electronic Engineering, Chengdu Technological University, Chengdu 610031, China; 2 School of Mechanical Engineering, Sichuan University, Chengdu 610065, Chinawzhijian5530@163.com (Z.W.);; 3 School of Pittsburgh Institute, Sichuan University, Chengdu 610065, China; 4 Department of ophthalmology, West China Hospital, Sichuan University, Chengdu 610041, China; 5 Med + X Center for Manufacturing, West China Hospital, Sichuan University, Chengdu 610041, China

**Keywords:** electronic nose system, gas sensor array, machine learning, SMOTE, feature extraction

## Abstract

Electronic nose systems are widely used in environmental monitoring and other related fields. In recent years, systems based on gas sensor arrays have attracted considerable attention. However, relying solely on improvements in gas-sensitive materials has struggled to break through the bottleneck in recognition accuracy. To address this challenge, this study designs and validates an integrated machine learning framework for enhanced gas identification in electronic nose systems. Specifically, (1) a Butterworth low-pass filter is combined with principal component analysis (PCA) to suppress sensor noise; (2) the synthetic minority over-sampling technique (SMOTE) is utilized for training set data augmentation to further enhance the classification accuracy of the support vector machine (SVM); and (3) the relationship between single-component and mixed-gas responses is analyzed to construct an artificial neural network (ANN) regression model. Experimental results demonstrate that the SMOTE-augmented, PCA-optimized SVM model achieves a recognition accuracy of 0.93 ± 0.08 for most target gases, representing improvements of 19% and 7% over decision tree and ANN classifiers, respectively, and that the ANN regression model attains a correlation coefficient of 99.55% between predicted and measured values in mixed-gas experiments. Overall, the construction and optimization of this system demonstrate significant practical value for intelligent gas identification and the development of advanced e-nose devices.

## 1. Introduction

Electronic nose systems based on gas sensor arrays are extensively used across diverse fields, including medical diagnostics, transportation safety, environmental monitoring, food quality assessment, agricultural cultivation, and livestock rearing [1,2,3]. In the medical field, electronic noses assist in early disease diagnosis by detecting specific biomarkers in exhaled breath. For transportation safety, they enable real-time alcohol monitoring for driver safety. In environmental monitoring, they are employed to assess air and water quality and to detect and classify volatile organic compounds (VOCs) [4,5]. Within the food industry, electronic noses facilitate automated and efficient food quality evaluation and flavor classification. In agriculture, they support early detection of plant pests and diseases, while in livestock farming, they are commonly used to monitor animal health and rearing-environment conditions. However, the limited recognition accuracy and low efficiency of gas sensors continue to pose significant barriers to their broader deployment [6].

To enhance recognition performance, traditional methods have focused on optimizing gas-sensitive materials, such as developing new sensing elements with improved noise immunity [7,8,9]. While these material-based strategies have achieved certain success, further progress has become increasingly constrained due to inherent performance ceilings. In this context, machine learning offers promising solutions for enhancing the intelligence and adaptability of electronic nose systems, particularly in data processing and information extraction [10,11].

Various innovative methods have been proposed for feature extraction and data representation. For instance, Guo et al. [12] proposed a new method that combines a Butterworth low-pass filter with PCA. Compared to the traditional PCA method, this approach can effectively solve the problem of high false alarm rate caused by noise. Similarly, Liu et al. [13] proposed a time correlation-based encoding scheme to address redundant features and synchronization issues in multi-sensor data. By transforming time series responses into image-like formats and applying deep neural networks, their method significantly improved recognition performance in alcohol classification tasks. Additional advances include non-parametric kernel modeling for non-linear features, Label-Consistent K-Singular Value Decomposition (L-KSVD), and Minimum Distance Increment Probability (MDIP), all of which demonstrate strong representational and classification capabilities [14,15].

Accurate gas composition classification is a core functionality of electronic noses. Bruno et al. [16] trained a multilayer ANN to improve the selectivity of metal oxide (MOX) sensors for detecting NH_3_, CH_4_, N_2_O, and air. Abbatangelo et al. [17] proposed a hybrid K-Nearest Neighbor–ANN (K-NN-ANN) model for beer gas classification, achieving improved precision. Liu et al. [18] introduced an ensemble learning framework combining random forest, Logistic Regression, and K-Nearest Neighbors (KNNs), significantly enhancing lung cancer detection. In addition, many scholars have performed relevant research on gas classification using electronic noses [19,20,21,22].

Despite the increasingly optimized performance of current gas recognition models, the problem of sensor drift remains severe. Environmental factors such as humidity, sensor aging, and mixed-gas interactions can cause significant deviations in sensor responses, compromising model stability and transferability. Moreover, online calibration remains difficult due to the scarcity of labeled drift samples. To address this, Zhu et al. [23] proposed a multi-task learning framework utilizing one-class drift calibration data, while Liu et al. [24] introduced an active sample selection strategy based on novel evaluation metrics. Additionally, several studies have proposed practical strategies for drift compensation under limited sample conditions [25,26,27].

Despite considerable advances in existing research, three key challenges remain: (1) noise suppression and dimension compression in feature extraction are difficult to coordinate, leading to insufficient robustness in feature representation; (2) the model’s classification accuracy is limited under small sample conditions; and (3) the interaction mechanism between single- and mixed-gas components lacks theoretical support, which hinders the continuous improvement in multi-component detection performance.

**Figure 1:**
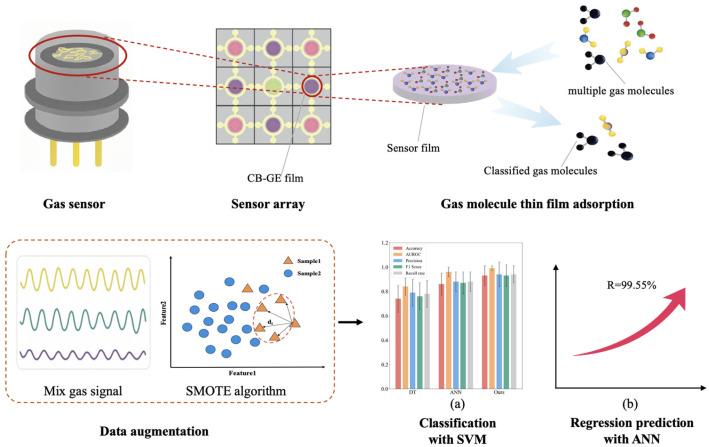
Flowchart of machine learning-driven gas identification methodology.

To address these challenges, our research focuses on systematically integrating and collaboratively optimizing the existing signal processing and machine learning technologies distinguished by three key design features, as shown in Figure 1. The gas to be detected is adsorbed through the sensing film of the sensor array in the encapsulated gas sensor and the signal is transmitted to the processing system. Firstly, a dual-stage feature engineering process combines Butterworth filtering, which is simple and efficient in eliminating noise, with PCA, which is more suitable for the dataset characteristics and the goal of dimensionality reduction, to preserve signal fidelity and optimize discriminative feature representation. Secondly, we developed a SMOTE-enhanced learning framework specifically engineered to ensure the representativeness of the generated samples; ultimately, the SVM uses high-quality balanced data to construct the optimal classification boundary, thereby improving the robustness and accuracy of gas classification. Thirdly, a systematic investigation into component interaction mechanisms between mixed gases and their pure counterparts establishes a research foundation for drift compensation in multi-gas environments.

Unlike earlier studies that applied PCA or SMOTE independently to address isolated issues such as high dimensionality or data imbalance, our research emphasizes stepwise optimization and synergistic enhancement. Through signal processing optimization, adaptive data expansion, and component relationship modeling, it solves the problems of high noise, small sample size, imbalance data, and redundant features in the electronic nose systems, demonstrating greater potential in overcoming the persistent limitations of conventional systems.

The remainder of this paper is structured as follows: Section 2 presents the proposed algorithms. Section 3 provides a detailed description of the dataset and the experimental evaluations. Finally, the conclusions are drawn in Section 4.

## 2. Methods

The dataset used in this study was collected using an array of 16 cross-sensitive metal oxide (MOX) sensors, collecting 58 time series samples under flow modulation conditions and covering multiple binary mixtures of acetone with ethanol (12 gas classes in total), with target gas concentrations ranging from 0.1 to 1 vol.%.

In this section, the gas data preprocessing and mining approaches proposed by us are elaborated. First, we describe the data preprocessing methods used in this study, including the filtering algorithm, feature extraction, and dimension reduction algorithm. Then, a machine learning algorithm for gas data classification is introduced. Finally, the proposed SMOTE data augmentation method is introduced in this subsection.

### 2.1. Gas Data Preprocessing Method

Raw sensor signals exhibit slow dynamics but are often contaminated by high-frequency noise from environmental and circuit sources, necessitating filtering for reliable identification. A third-order Butterworth low-pass filter was applied to smooth the raw sensor signals, which is determined by specific signal characteristics and the requirements of the integrated framework.

The Butterworth filter’s maximally flat passband allows effective high-frequency attenuation while preserving the essential shape and amplitude of the original signal, which is crucial for subsequent feature extraction. Furthermore, our multi-algorithm combination requires a preprocessing method with deterministic behavior and minimal parameter tuning to ensure stable input for downstream algorithms.

In contrast to other denoising methods (e.g., wavelet thresholding), while wavelet denoising excels at handling non-stationary signals and transient mutations, it requires complex selections of wavelet basis, decomposition levels, and threshold rules [28]. This multi-parameter adjustment brings uncertainty and complexity, making it less suitable for our integrated framework, which prioritizes stability and reproducibility over adaptive denoising capabilities.

Subsequently, a moving average filter was employed for further smoothing to mitigate residual random fluctuations and yield a stable baseline for feature extraction.

### 2.2. Adopted Feature Extraction Method

For feature extraction, the maximum amplitude of the low-frequency component for each sensor was selected as the primary feature. This metric robustly represents the steady-state sensor response and has been widely used in MOX sensor analysis due to its strong correlation with gas concentration and identity. While time domain characteristics offer a broad range of potential features, the maximum steady-state amplitude provided an optimal balance between discriminative power and stability for our classification task.

There may be strong correlations among data from various dimensions of gas sensors, which leads to redundancy of information. Ultimately, PCA was used for feature dimensionality reduction, primarily based on task objectives and data characteristics [29]. PCA extracts directions of maximum variance in the data via orthogonal transformation as principal components. These components can effectively eliminate redundancy and reduce dimensionality while preserving original information, thereby providing an optimal feature subspace for the SVM to construct a clear discriminative boundary. Moreover, there is a strong linear correlation among the response signals of the sensor array, and the data roughly follow a Gaussian distribution, which is exactly the form that PCA is good at handling. If the sensor data does not completely follow a Gaussian distribution, PCA still has considerable robustness. In contrast, independent component analysis (ICA) aims to recover statistically independent source signals, and its solution relies on non-Gaussian assumptions, with uncertainties in sequence and amplitude. For an electronic nose system with classification as the core task, PCA is more suitable due to its efficient computation and stable results.

### 2.3. Machine Learning-Based Classification Algorithms

The selection of machine learning models was driven by the characteristics of our dataset (small sample size and high dimensionality after feature extraction) and the specific tasks of classification and regression. Three adopted machine recognition algorithms were implemented: decision tree (DT) [30] and SVM [31] for gas classification, and ANN for regression analysis of mixture responses [32].

The SVM served as our primary classifier based on three principal considerations aligned with our experimental dataset. First, SVMs are particularly effective for small-to-medium-sized datasets similar to the one used in our experiment, as they aim to find the maximum-margin hyperplane that generalizes well, mitigating overfitting risk. Second, after PCA dimensionality reduction, our features resided in a lower-dimensional space where classes were more likely to be linearly separable or separable using the kernel trick. Third, compared to more complex ensemble methods, SVM offers a favorable balance between model complexity, computational efficiency, and predictive performance for our problem scale.

For comparison, a DT was implemented as a representative of simple, interpretable, and non-parametric models. However, its tendency to overfit noisy data without careful pruning makes it a challenging baseline. An ANN was also employed, primarily for its strong function approximation capability in modeling the regression relationship between pure and mixed-gas responses. Its use in the primary classification task was as a performance benchmark against the SVM.

### 2.4. SMOTE-Based Data Augmentation Algorithm

The SMOTE algorithm was employed to address the issue of limited data. This method generates synthetic samples without distorting the original feature relationships. Regardless of whether the original gas data points are linearly distributed or form clusters, the newly generated samples maintain the same distribution as the original data.

## 3. Results

### 3.1. Experimental Dataset

The experiment utilized the publicly available “Gas Sensor Array under Flow Modulation” dataset provided by Andrey Ziyatdinov et al. [33]. The dataset contains 58 time series samples collected by 16 metal oxide (MOX) gas sensors under flow modulation conditions, involving various binary mixtures of acetone and ethanol, which constitute 12 distinct gas classes.

The construction of this sensor array is detailed in the accompanying methodology. The array consists of 16 FIGARO TGS MOX sensors of five different models, configured into ten distinct sensing condition modes based on combinations of model type and operating temperature. These sensors are cross-sensitive; they are designed not for specific gas detection but to generate rich response patterns through surface reactions, thereby enabling the discrimination of complex gases via array-based pattern recognition [33]. Their detection range covers acetone and ethanol concentrations of 0.1, 0.3, and 1 vol.%.

Table 1 details the specific sample distribution of the 12 gas classes in the dataset (data source: [33]). As shown, the dataset includes 58 samples of pure ethanol, pure acetone, their binary mixtures, and air blanks. All measurements were completed within four days and divided into five batches (the ‘batch’ attribute) to minimize the impact of long-term sensor drift and environmental noise. The transient signals from each of the 16 sensors were recorded at a sampling frequency of 100 Hz, resulting in 7500 data points per sensor per 5 min measurement.

The detailed construction method of this dataset and its flow modulation strategy were designed to simulate the biological respiratory cycle, thereby improving early-stage gas detection performance.

We processed the dataset with a normalization method, which is an approach to data standardization that scales the data into the range of [0, 1]. After completing the above normalization based on minimum–maximum values, and since PCA and SVM are sensitive to the scale of features, we standardized the feature data. Specifically, for the normalized feature matrix, we calculated the mean and standard deviation of each feature dimension (i.e., each sensor or each principal component) and performed z-score normalization, so that the mean of each feature distribution was 0 and the standard deviation was 1.

### 3.2. Feature Extraction

#### 3.2.1. Frequency Domain and Time Domain Feature Extraction

In the experiment, two third-order Butterworth filters were used for feature extraction. A low-pass filter with a cutoff frequency of 0.01 Hz and a high-pass filter with a cutoff frequency of 0.07 Hz is used to obtain the component information of different frequency bands. These cutoff frequencies were determined through preliminary spectral analysis of the sensor baseline and dataset, which confirmed that the characteristic slow transient responses of the target gases reside below 0.01 Hz, while dominant noise components lie above 0.07 Hz. We empirically evaluated the impact of the low pass filter cutoff frequency on downstream classification accuracy. A sweep from 0.005 Hz to 0.05 Hz revealed that a cutoff of 0.01 Hz yielded the optimal balance, maximizing noise suppression while preserving the discriminative shape of the transient response.

#### 3.2.2. PCA Algorithm-Based Features Compression

**Figure 2:**
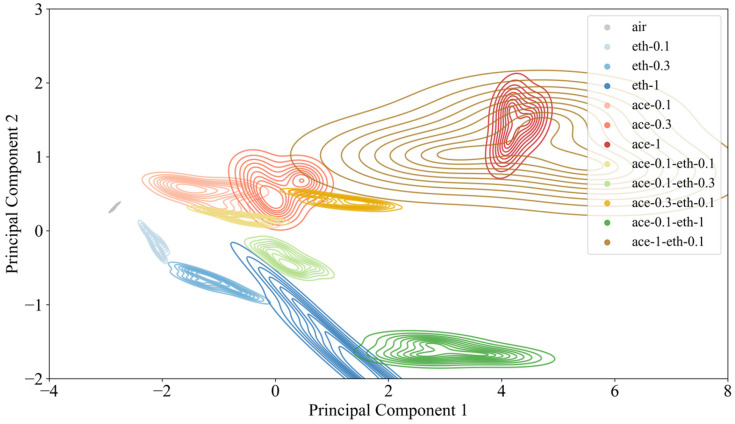
Gas features after the PCA dimensionality reduction.

Subsequently, the PCA algorithm was applied for feature dimensionality reduction. According to the specificity of the response signal of each gas sensor, the characteristics of each signal are extracted. Taking the largest value of the low-frequency signal component in each signal frequency domain as the feature, each sample has sixteen-dimensional characteristic information. Then, the PCA approach was employed to perform dimensionality reduction on the multidimensional features. The results of PCA are shown in Figure 2. It presents a two-dimensional PCA projection of the gas sensor data, where the horizontal and vertical axes represent Principal Component 1 (PC1) and Principal Component 2 (PC2). PC1 and PC2 explained 84.1% and 13.3% of the total variance of the data, respectively, and the cumulative variance contribution rate reached 97.4%. The scatter plot depicts distinct gas components through color coding: gray for air, blue for ethanol at varying concentrations, red for acetone at different concentrations, and yellow green for ethanol–acetone mixtures. Color intensity corresponds to gas concentration levels, with darker hues indicating higher concentrations.

The spatial distribution of data points reveals meaningful patterns in the reduced-dimensional space. Proximity between points (e.g., ‘ace-0.1’ and ‘ace-0.3’) indicates similarity in the original high-dimensional data, while distant points (e.g., ‘eth-1’ and ‘ace-1’) represent fundamentally distinct gas classes. The PC1 axis spans a broad range (−4 to 8), capturing the most significant variance in the dataset, which primarily reflects concentration-dependent variations. Notably, pure ethanol and acetone samples cluster in lower PC1 regions, while gas mixtures occupy higher PC1 regions, with a rightward shift observed with increasing concentration.

PC2 accounts for secondary variance patterns orthogonal to PC1, revealing a gas-type gradient from bottom to top: ethanol samples dominate the lower region, acetone samples the upper region, with their mixtures distributed intermediately. This vertical separation suggests PC2 effectively discriminates between different gas classes. Furthermore, mixture samples exhibit proximity to their dominant component—for instance, ‘ace-1-eth-0.1’ clusters nearer to ‘ace-1’, while equimolar mixtures (e.g., ‘ace-0.1-eth-0.1’) show greater affinity to the acetone component (‘eth-0.1’), suggesting an asymmetric influence of the constituent gases on the mixture response.

### 3.3. Data Augmentation and SVM-Based Classification Algorithm

#### 3.3.1. Classification Results of the Proposed Data Expansion Method

**Figure 3:**
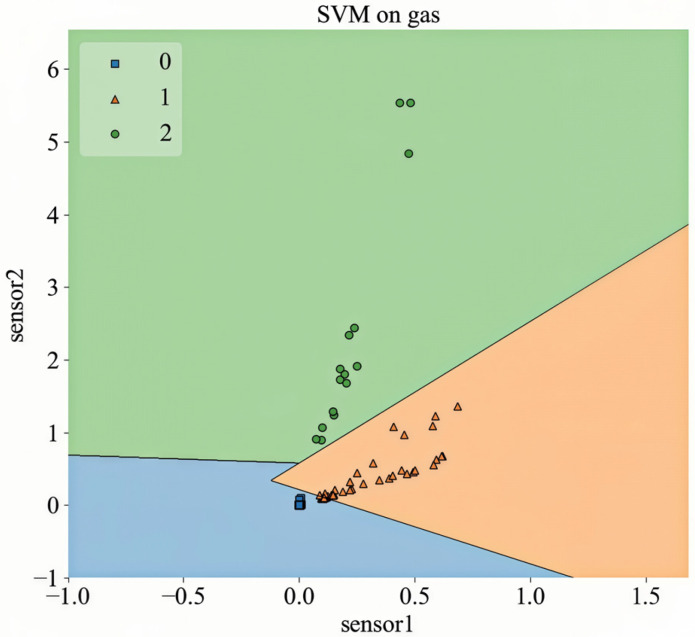
SMOTE algorithm fitting data for SVM classification.

Through comparative experiments, the optimal penalty parameter was identified as 20, and the radial linear basis function was selected as the kernel for the SVM model. The result of classifying the generated data by the SVM are shown in Figure 3. It can be seen from the figure that the characteristics of various gases are well classified. For example, the measured signal values of air are small, so the sample points basically coincide. The sample point distribution of acetone is approximately linear, while that of ethanol is concentrated.

**Figure 4:**
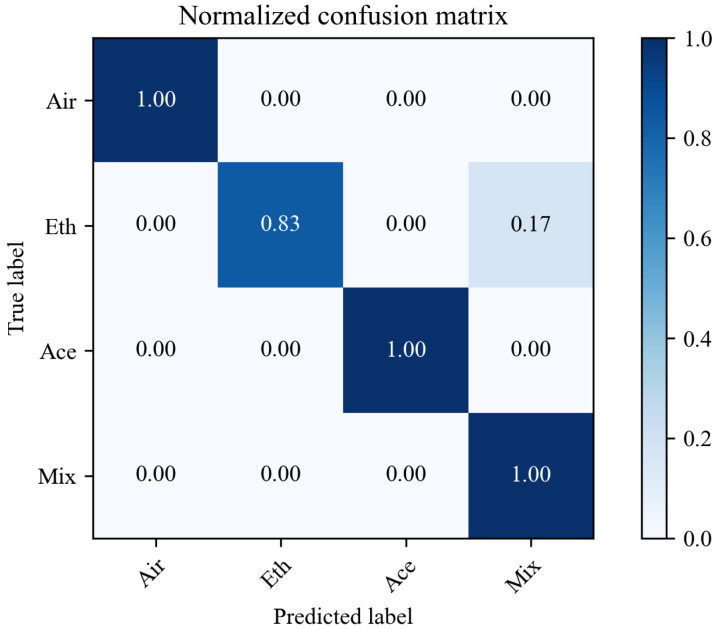
Confusion matrix of the results.

Furthermore, based on the established SVM model, the test data were classified into four categories (air, ethanol, acetone, and mixture), and the confusion matrix was calculated. The calculated confusion matrix is shown in Figure 4. Obviously, except for the misclassification of ethanol, all other gases have 100% accurate classification results.

#### 3.3.2. Comparison of Machine Learning Algorithms

To evaluate the effectiveness of gas classification using the proposed method based on SMOTE and SVM after data preprocessing, comparative experiments were conducted against DT and ANN algorithms.

Although KNN, random forest (RF), gradient boosting, and extreme gradient boosting (XGBoost) are frequently used in electronic nose research, the limited size of our small sample dataset (N = 58) imposes inherent limitations on such common ensemble learning methods. The distance metric relied upon by the KNN algorithm in a sparse feature space is unreliable and sensitive to noise; RF suffers from excessive correlation among sub-trees due to insufficient data diversity, thereby undermining its advantage in variance reduction; and boosting algorithms like XGBoost, whose iterative correction mechanisms are prone to overfitting random noise in the data rather than learning true gas patterns, have poor generalization ability. In contrast, simpler models such as DT and ANN achieve a better balance between accuracy and computational efficiency under these circumstances.

To ensure a robust statistical assessment, we performed twenty repeated runs of fivefold cross-validation. In each run, the dataset was randomly divided into five equal-sized subsets, orderly using one as the test set and the other four for training. This procedure yielded a total of 100 independent evaluations, effectively minimizing the risk of bias from any single data split and providing a reliable estimate of model performance. The experimental results are summarized in Table 2.

The average classification accuracies achieved by the DT and ANN were 0.74 ± 0.11 and 0.86 ± 0.09, respectively. In contrast, the proposed model, which integrates SMOTE-based over-sampling and feature extraction techniques, achieved a significantly higher accuracy of 0.93 ± 0.08.

Furthermore, the SVM model demonstrated superior performance across multiple evaluation metrics, including Accuracy, Area Under the Receiver Operating Characteristic Curve (AUROC), Precision, F1-score, and Recall rate, outperforming both baseline models. These results validate the effectiveness and robustness of the proposed approach for gas classification tasks.

Our model is optimized through Butterworth filters, PCA, and SMOTE. Butterworth filter effectively removes high-frequency noise while maintaining signal integrity, enhancing SVM’s dependence on well-defined support vectors, PCA enhances the SVM’s ability to construct optimal hyperplanes in low-dimensional space through dimensionality reduction, SMOTE alleviates class imbalance by generating synthetic minority samples, enabling SVM to learn more fair decision boundaries. In contrast, the tendency of DT to overfit and the requirement of ANNs for large tuning and large data volumes make them perform relatively poorer when noise, dimensionality, and imbalances are prominent.

### 3.4. Response of Gaseous Mixture Prediction

**Figure 5:**
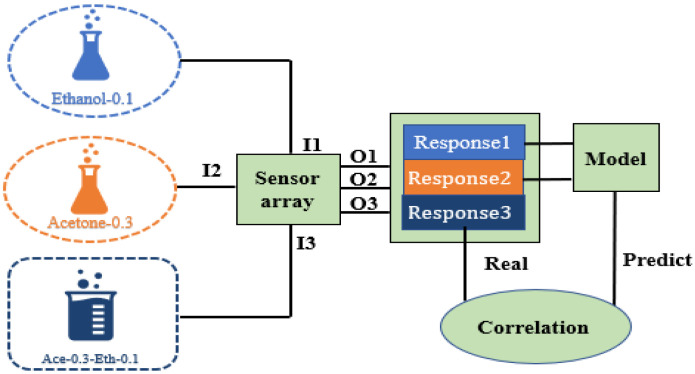
Schematic diagram of the gas prediction regression model establishment.

To resolve the problem of data drift caused by mixed gases, the correlation between single-gas and mixed-gas responses is studied in this subsection. The main research route is shown in Figure 5. An ANN regression prediction model is established to describe the mapping relationship between single-gas and mixed-gas responses.

**Figure 6:**
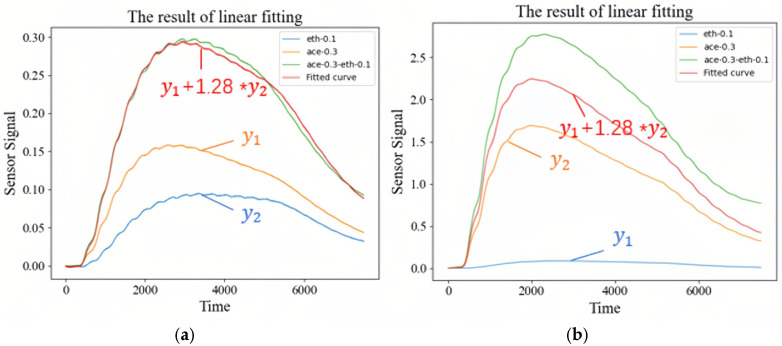
Response relationship between single-gas and mixed-gas: (**a**) single-gas; (**b**) mixed-gas.

First, it is assumed that there is a linear relationship between the responses of single-gas and mixed-gas, and that the response of mixed-gas is a linear combination with a ratio of 1:1.28 (ethanol/acetone). The fitting result is shown in Figure 6a. However, when this proportional coefficient is used to fit the measured responses of gas sensors in other channels, it is found that the errors are quite different. The results are shown in Figure 6b. Although the fitted curve and the actual response “ace-0.3-eth-0.1” have a similar trend, the amplitude difference is large. Therefore, the relationship between single-gas and mixed-gas is not a linear superposition relationship.

**Figure 7:**
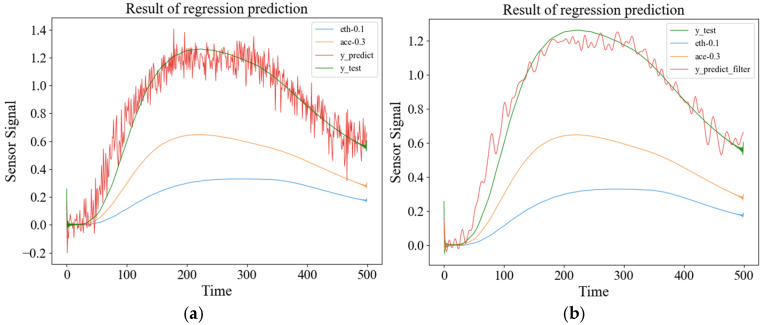
Prediction results and smoothed results of the ANN: (**a**) predicted results by ANN; (**b**) smoothed results.

Figure 7a shows a linear fitting of the correlation between single-gas and mixed-gas responses used to train the neural network and generate the regression model. The test results are shown in the figure. According to the test results, the scores were calculated. The correlation between the predicted results and the actual measured results is strong, with a calculated correlation coefficient of 97.83%.

It can be seen from Figure 7a that there are many burrs in the predicted signal. To smooth it, a low-pass filter is used, and the filtered result is shown in Figure 7b. Comparing the filtered curve with the actual test signal, the correlation coefficient is 99.31%, which is improved compared with the unfiltered result. Furthermore, the polynomial fitting method is used to fit the prediction results of ANN. The final calculated correlation coefficient is 99.55%, which improves the accuracy compared with that before fitting.

Sensor drift involves complex, long-term temporal changes in sensor baseline and sensitivity due to factors like aging, poisoning, and environmental fluctuations (e.g., humidity), which are not represented in this dataset. Addressing drift requires dedicated long-term calibration data and domain adaptation techniques.

### 3.5. Discussion on Model Robustness and Generalization

Although the proposed ANN regression model achieves 99.55% under controlled experimental conditions, its long-term reliability in practical deployments remains a critical consideration. Metal oxide gas sensors are inherently susceptible to long-term drift and sensor aging, caused by factors such as sensitive material degradation, ambient temperature and humidity fluctuations, and prolonged exposure to complex gas mixtures. These phenomena can lead to non-linear shifts in sensor response patterns, potentially compromising the predictive accuracy of the model over time.

To enhance the system’s robustness against such variations, two main strategies are proposed for future optimization. First, the integration of Calibration Transfer techniques, such as Domain Adaptation or Transfer Learning, could be employed to map the response characteristics between the initial calibration state and the current drifted state. This would ensure model consistency without the need for exhaustive and labor-intensive re-calibration. Second, incorporating environmental compensation terms—by using real-time temperature and humidity data as auxiliary inputs—could further mitigate interference from fluctuating ambient conditions.

Future work will focus on validating the framework using long-term datasets collected in diverse field environments, including more complex VOC mixtures involving more than three gases. By implementing drift-robust feature extraction and adaptive re-calibration mechanisms, the proposed system can achieve higher operational stability and broader applicability in real-world gas monitoring scenarios.

## 4. Conclusions

This study proposes a machine learning framework that effectively tackles noise interference, small and imbalanced datasets, and sensor drift in mixed-gas environments. The approach combines Butterworth filtering with PCA for optimized feature extraction, while leveraging SMOTE-based data augmentation to enhance classification robustness. Compared with the traditional DT algorithm, the model improves classification accuracy by 19% and AUROC by 15%. Additionally, drift compensation is supported by high-correlation regression modeling of up to 99.55%. Although the framework demonstrates superior performance in binary and multi-class gas classification tasks, the current study is limited by the small dataset size, manual feature extraction, and its adaptability to complex real-world scenarios. Furthermore, the high correlation achieved by the ANN regression model for binary ethanol–acetone mixtures was obtained under limited, short-term laboratory conditions and should not be directly equated with a general sensor drift compensation solution. In the future, cross-domain data or supplementary databases of more complex VOC mixtures involving more than three gases in real environments can be used to make up for the shortcomings of the small-scale training set, further verifying the robustness and adaptability of the model framework. In addition, attempts will be made to use automatic feature extraction methods such as Long Short-Term Memory networks or Convolutional Neural Networks. This study will provide a practical and efficient technical framework for embedded electronic nose systems in fields such as medical diagnostics, environmental monitoring, and food safety.

## Figures and Tables

**Table 1:** Gas sensor array dataset composition.

Label	Concentration (vol.%)	Number of Samples
ETH-0.1	0.1	6
ETH-0.3	0.3	4
ETH-1.0	1.0	5
ACE-0.1	0.1	6
ACE-0.3	0.3	6
ACE-1.0	1.0	3
ACE-0.1 + ETH-0.1	0.1 (ACE) + 0.1 (ETH)	4
ACE-0.1 + ETH-0.3	0.1 (ACE) + 0.3 (ETH)	5
ACE-0.3 + ETH-0.1	0.3 (ACE) + 0.1 (ETH)	5
ACE-0.1 + ETH-1.0	0.1 (ACE) + 1.0 (ETH)	3
ACE-1.0 + ETH-0.1	1.0 (ACE) + 0.1 (ETH)	3
Air	-	8

**Table 2:** Comparative experimental results.

Model	Accuracy	AUROC	Precision	F1 Score	Recall Rate
DT	0.74 ± 0.11	0.84 ± 0.07	0.79 ± 0.11	0.76 ± 0.11	0.78 ± 0.11
ANN	0.86 ± 0.09	0.96 ± 0.04	0.88 ± 0.08	0.87 ± 0.09	0.88 ± 0.08
Ours	0.93 ± 0.08	0.99 ± 0.02	0.94 ± 0.10	0.93 ± 0.09	0.94 ± 0.07

## Data Availability

The original contributions presented in this study are included in the article. Further inquiries can be directed to the corresponding authors.

## Abbreviations

The following abbreviations are used in this manuscript:

PCA	Principal Component Analysis
SMOTE	Synthetic Minority Over-Sampling Technique
ICA	Independent Component Analysis
ANN	Artificial Neural Network
KNN	K-Nearest Neighbors
DT	Decision Tree
SVM	Support Vector Machine
